# Comparison of behind-the-ear vs. off-the-ear speech processors in cochlear implants: A systematic review and narrative synthesis

**DOI:** 10.1371/journal.pone.0318218

**Published:** 2025-01-27

**Authors:** Muhammed Ayas, Jameel Muzaffar, Veronica Phillips, Manohar Bance

**Affiliations:** 1 College of Health Sciences, University of Sharjah, Sharjah, United Arab Emirates; 2 Department of Clinical Neurosciences, University of Cambridge, Cambridge, United Kingdom; 3 Cambridge Hearing Group, University of Cambridge, Cambridge, United Kingdom; 4 Research Institute of Medical and Health Sciences (RIMHS), University of Sharjah, Sharjah, United Arab Emirates; 5 Department of ENT, University Hospitals Birmingham NHS Foundation Trust, Birmingham, United Kingdom; 6 Department of Applied Health Sciences, University of Birmingham, Birmingham, United Kingdom; 7 Medical Library, University of Cambridge School of Clinical Medicine, Cambridge, United Kingdom; 8 Department of ENT, Cambridge University Hospitals NHS Foundation Trust, Cambridge, United Kingdom; Kasturba Medical College Mangalore / Manipal Academy of Higher Education, INDIA

## Abstract

**Background:**

Cochlear implants (CI) with off-the-ear (OTE) and behind-the-ear (BTE) speech processors differ in user experience and audiological performance, impacting speech perception, comfort, and satisfaction.

**Objectives:**

This systematic review explores audiological outcomes (speech perception in quiet and noise) and non-audiological factors (device handling, comfort, cosmetics, overall satisfaction) of OTE and BTE speech processors in CI recipients.

**Methods:**

We conducted a systematic review following PRISMA-S guidelines, examining Medline, Embase, Cochrane Library, Scopus, and ProQuest Dissertations and Theses. Data encompassed recipient characteristics, processor usage, speech perception, and non-audiological factors. Studies were assessed for quality and risk of bias by using Newcastle-Ottawa Scale (NOS).

**Results:**

Nine studies involving 204 CI recipients, with a mean age of 49.01 years and 6.62 years of processor use, were included. Audiological results indicated comparable performance in quiet environments, with a slight preference for OTE in noisy conditions. For non-audiological factors, OTE processors excelled in comfort, handling, and aesthetics, leading to higher satisfaction. More data on medical complications and long-term implications is needed.

**Conclusion:**

OTE processors may offer comparable performance to BTE processors in certain conditions, though not universally across all audiological outcomes. Interpretation depends on settings, processor generation, and testing paradigms. However, non-audiological factors might favour OTE. Understanding current literature may guide professionals in selecting suitable processors for CI recipients.

## Introduction

Cochlear implants (CI) have revolutionized auditory rehabilitation for individuals with severe to profound sensorineural hearing loss (SNHL), principally by restoring speech perception [1]. Despite the success of CIs in improving hearing abilities, there remain challenges to enhance the overall recipient experience as well further improving speech perception abilities, especially in the presence of background noise [2]. An important clinical aspect of the CI pathway is the selection of appropriate speech processors. CI manufacturers have historically varied designs of sound processors to a similar degree to implantable components. However, currently available speech processors may be broadly divided into behind-the-ear (BTE) and off-the-ear (OTE) processors [3, 4]. The main body of BTE speech processors, i.e. housed behind the ear, contains the microphone and processor, which is then connected to the receiver-stimulator with a cable and coil. On the other hand, OTE processors embed the microphone and processor within one unit, typically positioned retro-auricularly on the implant magnet for stimulation, without any additional coil or cables, thus providing a discrete wireless appearance.

CI speech processors are an essential component and play an important role in translating acoustic sounds into electrical signals that can then be presented to the intracochlear electrodes for stimulation of the residual spiral ganglion cells [5]. Traditionally BTE speech processors have been the standard choice for CI recipients. Initial devices utilised body worn processors, but these were ultimately superseded by BTEs. However, recent advances in processor technology have introduced OTE devices as an alternative option to BTE.

OTE processors were introduced to offer recipients an additional option of a wireless device with both microphone and coil embedded in one unit. Currently there are two manufacturers offering commercial OTE options: MED-EL® (Rondo-1, Rondo-2. Rondo-3) and Cochlear® Ltd (Kanso-1, Kanso-2). One of the reported challenges in OTE is that the microphone positioning in OTE processors may be less optimal than in BTE devices for speech perception, particularly in noisy environments. Although some studies report no significant differences in speech perception between OTE and BTE processors [6–8], the limited number and varied methodologies of these studies leave room for uncertainty. This underscores the need for further evaluation of OTE processors, not only for speech understanding but also for device handling, comfort, and ease of use [4, 9, 10]. As a result, BTE devices remain more commonly used in clinical practice, likely due to familiarity and established use. While both BTE and OTE options are available, further research is needed to clarify how factors like familiarity, reliability, and patient preferences shape clinician recommendations and patient choices.

This systematic review aims to comprehensively examine and compare the effects of BTE and OTE speech processors on both audiological performance in quiet and noisy conditions, as well as non-audiological factors such as ease of use, device handling, medical complications, and others. Specifically, this study evaluates the performance and subjective ratings for both processors in adult users only. Additionally, it describes the evaluation of OTE processors by CI users who already had experience with a BTE processor. This synthesis will provide clinicians and CI recipients with evidence on the use of OTE and BTE technology to make informed decisions.

## Methods

We conducted a systematic review based on the guidelines outlined in the preferred reporting items for systematic reviews and meta-analyses (PRISMA-S). The review was prospectively registered on the PROSPERO prior to the commencement of the literature search on July 10, 2023. The full protocol can be accessed via the link- https://www.crd.york.ac.uk/prospero /display_record.php?RecordID = 443972.

### Study inclusion criteria

The literature search followed the PICO:

Population: Adult CI recipients

Intervention: OTE devices

Comparison: BTE devices

Outcome(s): Audiological and non-audiological performance

No restrictions were placed on study design for inclusion. Studies carried out as cross-sectional studies, longitudinal, experimental, quasi-experimental and observational studies were all included in the review. However, studies comparing BTE and OTE processors in children were excluded. Additionally, case reports, editorials, and studies not conducted in English or lacking an English translation were excluded.

### Search strategy

The following databases were searched: Medline (via Ovid), Embase (via Ovid), Cochrane Library, Scopus, and ProQuest Dissertations and Theses were searched from inception to January 2024 by VP, a specialist clinical librarian. The search strategy was peer-reviewed by two librarian colleagues using the peer review of electronic search strategies (PRESS) checklist [11] and evaluated against the PRISMA-S guidelines [12] (See S1 File). Databases were searched by VP separately, rather than multiple databases being searched on the same platform. The search syntax was adapted for each database, and to account for variation between thesaurus terms or controlled vocabulary across each database. For example, for the database search in Medline (via Ovid) were as follows.

((cochlea* or auditory) adj5 (implant* or prosthe*)).ti,ab,kw. OR Cochlear Implants/ or Cochlear Implantation/

AND

("speech process*" or "sound process*").ti,ab,kw. or exp signal processing, computer assisted/

AND

("off the ear*" or OTE or ("behind the ear*" or BTE) or (microphone location or (entrance adj4 canal) or (top adj4 pinna))).ti,ab,kw.

All other databases searches have been presented in the supplementary material (See S2 File). Results were imported to Endnote 20 by VP for deduplication, using the method outlined by Bramer et al. [13]. The searches were rerun prior to submission to include any papers published between the initial searching, and submission for peer review.

### Study selection

The outcomes of the database search were transferred to the Rayyan web-based software (https://www.rayyan.ai) for independent and blinded eligibility screening. Duplicate studies were identified and removed using the Rayyan software. Subsequently, two authors (MA and JM) independently screened titles. Abstracts of included titles were then screened, and full-text articles were shortlisted and extracted by MA and JM. The reference lists of all full text articles were further screened to identify any studies not highlighted by database searches. Discrepancies raised during the process of article screening were resolved through consensus by a third author (MLB). Finally, articles which met the inclusion criteria were presented for full text review and data extraction.

### Data extraction

The findings were recorded on a data collection protocol sheet, tailored specifically for this study by MA and cross verified by JM and MLB. The following information data extracted from the included studies: authors, year of publication, study location, sample size and characteristics, age of included subjects, aetiology, number of implanted ears, duration of the speech processor usage, speech perception results for BTE and OTE, non-audiological outcomes such as device handling, comfort, cosmetics, and overall satisfaction along with self-assessment. Missing data in the included studies were handled by identifying and documenting areas where data were not reported, such as follow-up adequacy, outcome assessment, and selection of non-exposed groups.

### Quality assessment

Studies were assessed for quality and risk of bias by the reviewers using Newcastle-Ottawa Scale (NOS) by MA and JM. The NOS scale judges the quality of included studies in three areas: selection, comparability, and outcome. Additionally, the scale assesses the following: control cohort, the number of session (the length/follow up), and outcomes measures (objective or self-reported). The quality of the studies may be judged as either good (low risk), fair (high risk), or poor (very high risk) by awarding stars in each domain accordingly with NOS guidelines [14]. Discussion and consensus were reached between the authors (MA and JM) when there was a discrepancy in the ratings assigned to studies.

## Results

### Source of evidence

**Fig 1:**
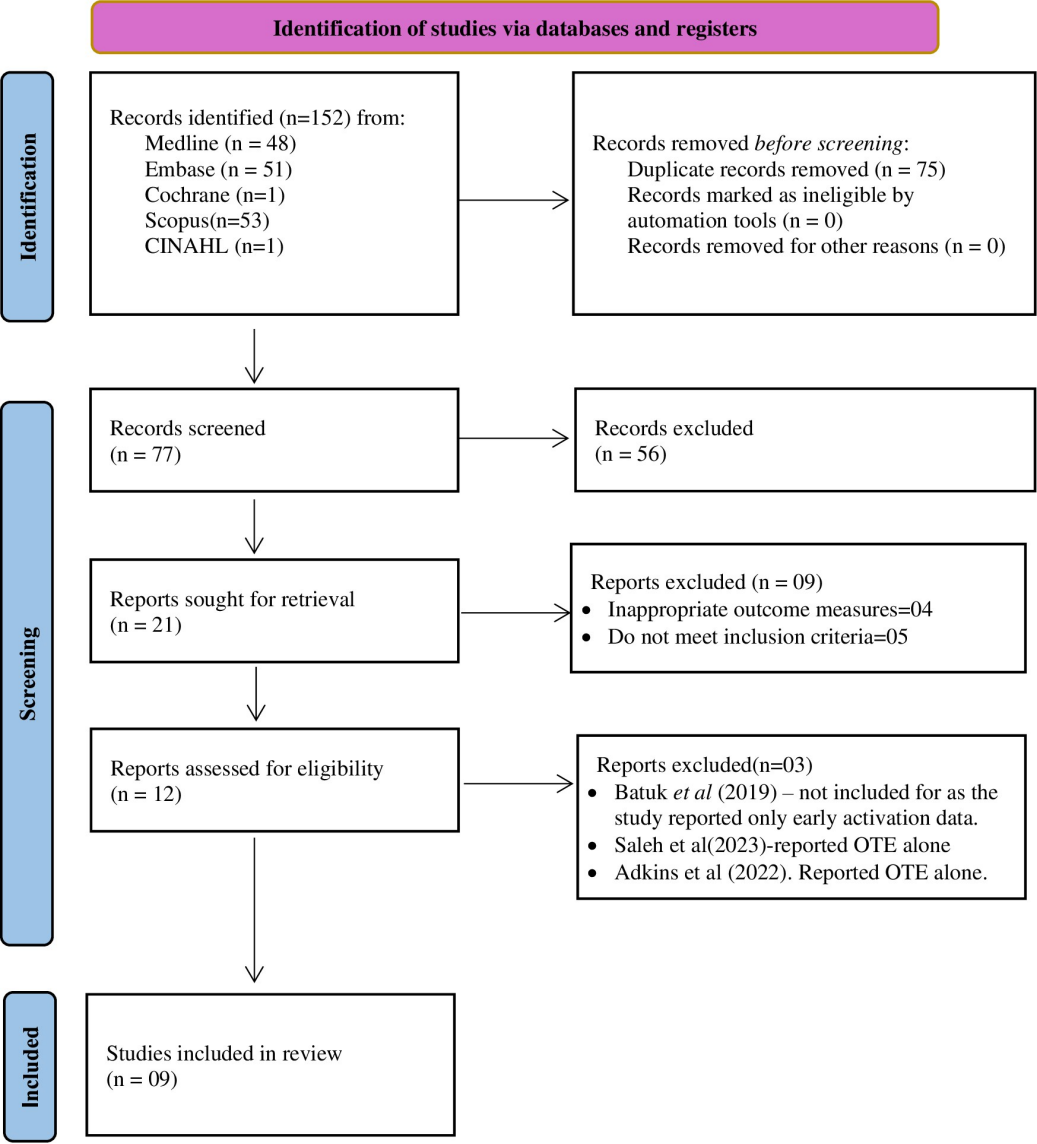
PRISMA flowchart for study identification and selection.

A total of 152 studies were identified through the search. Of these, 75 duplicate records were excluded. Abstracts for the remaining 77 articles were screened, and 56 were excluded because they did not meet the inclusion criteria. Subsequently, 21 full-text records were retrieved, and 9 more studies were excluded due to inappropriate outcome measures as they involved modelling studies or laboratory-based experiments which did not meet the predefined inclusion criteria for this review. Twelve records were assessed for eligibility, and three more studies were excluded because they reported only OTE data. The remaining 9 full-text articles were included in the review, and data were extracted. The screening, selection, and inclusion processes are illustrated in the PRISMA chart (Fig 1).

### Description of the studies

Two hundred and four CI recipients participated across the 9 studies reported in this review with an average age of 49.01 years (range: 30.59–61 years). All the studies utilised a prospective study design. The average speech processor usage across the groups were 6.62 years predominantly unilateral CI users. The BTE processors reported in the studies were of N5, N6, N7 versions from Cochlear® Ltd and Opus 2 and Duet processors from MED-EL®. OTE processors used in the studies were Kanso-1, Kanso-2 from Cochlear® Ltd and Rondo-1 from MED-EL®. Studies were conducted in South America-Brazil [15], Europe- Belgium [16], Germany [17, 18], Switzerland [19], Turkey [4] and Asia Pacific-Australia [20–22].

No reported studies were found from North America, the Middle East or Africa. It is important to highlight that no date restrictions were applied to the initial search strategy, thus supplementing the novelty and relevance of the studies included in this review. Table 1 summarizes the individual characteristics of the included studies. Table 2 summarizes the key research questions main findings for each study.

**Table 1:** Characteristics of the included studies.

	Author/Year	Location	Age (Mean)	Sample Size	Device configuration			BTE processor		OTE processor		Audiological tests					Non-Audiological Factors					Self-Assessment
SI					Unilateral	Bilateral	Bimodal	Cochlear	Medel	Cochlear	Medel	Quiet	Noise	Adaptative	Speech test Settings	SFAT	Medical Complications	Device Handling	Retention	Comfort	Overall satisfaction	
**1**	**Jones et al. 2023 [22]**	Australia	61	22	4	15	3	N7	0	Kanso-2	0	NR	AuSTIN- OTE better	AuSTIN- OTE better	S0N0 & S0Nrearhemi (Speech fixed& Noise varied) Spatially separated	NR	NR	OTE preferred over BTE	NR	OTE preferred over BTE	satisfied with OTE/same as BTE	SPEQ
**2**	**Pinheiro et al. 2021 [15]**	Brazil	35.3	51	27	11	13	N5	0	Kanso-1	0	HINT = No difference	HINT = OTE better	HINT = OTE better	S0N0 S0adapat N0 (Speech varied & Noise fixed) Co-located	NR	NR	NR	NR	NR	NR	NR
**3**	**Bayri et al. 2020 [4]**	Turkey	30.59	27	20	0	7	N6	0	Kanso-1	0	Turkish CNC test = No difference	Turkish Matrix test = OTE better	Turkish Matrix test = OTE better	S0N0 S0NIL S0NCL S0N180 S180N180 (Speech varied & Noise fixed) Co-located & Spatially separated	Yes	NR	NR	NR	NR	NR	NR
**4**	**Wesarg et al. 2018 [18]**	Germany	43.8	16	2	14	0	N5 & N6	0	Kanso-1	0	FMT = No difference	OST = No difference	OST = No difference	S0N0 S0NIL S0NCL S0N180 (Speech varied & Noise fixed) Co-located & Spatially separated	Yes	NR	NR	NR	NR	NR	NR
**5**	**Dazert et al. 2017 [17]**	Germany	56.1	41	NR	NR	NR	0	Opus2	0	Rondo-1	FMT = No difference	OST = No difference	NR	NR	NR	Redness, Irritation, itching and Pain with OTE	OTE preferred over BTE	NR	OTE preferred over BTE	satisfied with OTE/same as BTE	HISQUI = OTE Similar to that of BTE
**6**	**Mauger et al. 2017 [21]**	Australia	56.25	20	4	7	9	N5 & N6	0	Kanso-1	0	Australian CNC words = No difference	BKB = No difference	BKB = No difference	S0N0 S0Nrearhemi (Speech fixed& Noise varied) Co-located & Spatially separated	NR	NR	OTE preferred over BTE	No report of concerns	OTE preferred over BTE	OTE preferred over BTE	NR
**7**	**Wimmer et al. 2015 [19]**	Switzerland	52	12	12	0	0	0	Opus2	0	Rondo-1	NR	NR	OST = No difference	S0N0 S0NIL S0NCL S0N180 (Speech varied & Noise fixed) Co-located & Spatially separated	NR	NR	NR	NR	NR	NR	NR
**8**	**Távora-Vieira et al. 2015 [20]**	Australia	58.4	5	5	0	0	0	Duet	0	Rondo-1	CUNY- No difference	BKB-SIN- No difference	NR	S0N0 (Speech fixed& Noise fixed) Co-located	NR	NR	OTE preferred over BTE	NR	OTE preferred over BTE	OTE preferred over BTE	SSQ = OTE Similar to that of BTE
**9**	**Mertens et al. 2014 [16]**	Belgium	48.2	10	10	0	0	0	Opus2	0	Rondo-1	NR	LIST- No difference	LIST- No difference	S0N0 (Speech varied & Noise fixed) Co-located	Yes	NR	OTE preferred over BTE	Retention issues(44%)	OTE preferred over BTE	OTE preferred over BTE	HISQUI,SSQ,SHQ- Superior with OTE

*Note*: NR- Not reported;N5,N6-Speech processor from Cochlear Ltd; SFAT-sound field Aided threshold, BTE-behind The Ear; OTE- Off The Ear; A-CNC- Australian consonant-nucleus-consonant, AuSTIN- Australian Speech Test In Noise; BKB-SIN- Bamford-Kowal-Bench- Speech in noise; OST- Oldenburg Sentence Test; CUNY- City University of New York; FMT- Freiburg Monosyllables test; HINT- Hearing in Noise Test; LIST- Leuven Intelligibility Sentences; T-CNC- Turkish consonant-nucleus-consonant; TMT- Turkish Matrix test; SPEQ-Sound Processor Experience Questionnaire; HISQUI-Hearing Implant Sound Quality Index; SSQ-Speech, Spatial, and Qualities; SHQ- Spatial Hearing Questionnaire; > greater than.; S0N0-speech and noise from front; S0NIL-speech from front and noise form ipsilateral side of CI; S0NCL-speech from front and noise form contralateral side of CI; S0N180 -speech from front and noise from back; S180N180-speech and noise from back; S0N3-speech from the front and 4-talker babble noise from 3 speakers equal to 12-talker babble; S0Nrearhemi- speech from front and 4-independent talkers presented from four locations roving across 7-speakers in the rear hemispheric field; Co-located- Speech and noise from Front(Diotic); Spatially separated-Speech and noise coming from different angles(Dichotic).

**Table 2:** Key research questions and summary for each study.

Study	Key research Question	Summary of main findings/results
**Jones et al. 2023 [22]**	To investigate the hearing performance of OTE (Kanso-2) processor using spatial noise reduction features.	The study reported the efficacy of ForwardFocus, a spatial noise reduction setting in the OTE (Kanso-2) processor. Speech understanding in quiet conditions was matched or improved with ForwardFocus on the Kanso-2 compared to the Kanso-1. However, in more challenging noisy situations, the BTE (Nucleus-7) performed better than the Kanso-2. This difference was attributed to the placement of the implant receiver-stimulator due to surgical preference, which may influence the location of the microphone. These findings provide valuable insights for clinicians during processor selection and counselling.
**Pinheiro et al. 2021 [15]**	To compare the speech performance in CI users using OTE (Kanso-1) and BTE processors(N5)	The study compared two different generations of speech processor technologies: the older BTE processors (N5) and the newer OTE (Kanso-1). Results revealed superior performance by the OTE (Kanso-1) over the BTE (N5) processors in noisy conditions, with an 8.1% difference in sentence scores. OTE processors showed better SNR and higher sentence recognition scores among CI users. Kanso, with SCAN technology and automatic microphone directionality, offered better performance in various conditions compared to the N5, which has two omnidirectional microphones. It should be recognised that these variables significantly contaminate the comparisons.
**Bayri et al. 2020 [4]**	To compare speech in quiet and noise performance of adult CI users using OTE (Kanso-1) and BTE (N6) processors. Further compare the impact of microphone location on speech performance.	Study findings suggest that there was no significant difference (p > 0.05) in sound field aided thresholds (SFAT), speech discrimination scores, or intelligibility in noise between the two processors, indicating comparable performance between OTE and BTE processors
**Wesarg et al. 2018 [18]**	To understand the effect of dual microphone positioning in OTE (Kanso-1) processors by comparing the speech performance with that of BTE processors.	Results indicated comparable scores on word recognition and speech reception thresholds in quiet and noise with OTE processors. Adaptive directional microphone showed significant improvement in spatially separated sources. OTE processors offer advantages over BTE without compromising speech performance.
**Dazert et al. 2017 [17]**	To study the effect of OTE (Rondo-1) processors on speech perception abilities of the experienced CI users	Speech perception and subjective sound quality assessed at three intervals (0,1 and 6 months) showed no significant differences between OTE and BTE processors., with high user satisfaction reported for the OTE group. The study suggests that OTE is a safe and effective alternative to traditional BTE processors with comparable speech perception and user comfort.
**Mauger et al. 2017 [21]**	To investigate the clinical outcome and subjective ratings of OTE (Kanso-1) processor in comparison with BTE(N6) speech processor.	No significant differences were found between OTE and BTE for tests in quiet and co-located speech in noise with the standard microphone. However, when comparing processor types within Zoom, Beam, and SCAN settings, BTE (N6) performed better than OTE(Kanso-1), with a mean difference in SRT of 1.4 dB, 2.0 dB, and 1.4 dB, respectively. With the default SCAN program, OTE demonstrated significant improvements in co-located and spatially separated noise compared to standard directionality and single-microphone programs. Furthermore, CI recipients rated OTE higher in comfort, appearance, ease of use, music, and overall hearing performance.
**Wimmer et al. 2015 [19]**	To study effect of OTE (Rondo-1) processors in speech intelligibility in noise	Study findings show no statistically significant differences in different spatial configurations. However, when noise was presented from the back, BTE performed better than OTE. This indicates that if OTE are positioned further behind the ear, recipient may face increased challenges in noisy situations.
**Távora-Vieira et al. 2015 [20]**	To compare the objective and subjective outcomes with EAS BTE vs OTE (Rondo-1) processor combined with an ITE hearing aid	Results indicated improved speech understanding in quiet and noise with BTE processor and were stable after upgrading to OTE + ITE hearing aid. Subjective assessments indicated higher perceived hearing ability with OTE, with showing higher quality and spatial scores
**Mertens et al. 2014 [16]**	To evaluate the hearing performances of SSD CI recipients using BTE and OTE(Rondo-1) processors.	Results showed comparable hearing related outcomes, sound localization, speech quality and tinnitus reduction between OTE and BTE processors. The position of the microphone in OTE did not affect the performance. After a short period of usage, 80% of the recipients preferred OTE, indicating its feasibility as an upgrade option for long-term BTE processor users.

*Note*: OTE- Off the ear; BTE- Behind the ear; EAS- Electro acoustic stimulation; SSD- single side deafness; N5-Cochlear speech processor; SFAT (Sound field aided thresholds); SCAN- Automatic scene classifier

### Audiological outcomes

The audiological factors explored in the studies cover a range of performance measures for both BTE and OTE devices. Testing was performed in both quiet and in noise conditions. A wide array of speech tests were conducted, including the Hearing in Noise Test (HINT), Turkish Consonant-Nucleus-Consonant (CNC) test, City University of New York (CUNY) Sentence lists, Freiburg Monosyllables test (FMT), Australian CNC words, Turkish Matrix test (TMT), Oldenburg Sentence Test (OST), Bamford-Kowal-Bench Speech in noise (BKB-SIN) sentences, Leuven Intelligibility Sentences Test (LIST), AuSTIN- Australian Speech Test In Noise Tests. The extracted data from these studies indicates an intriguing preference for OTE devices across various speech performance testing conditions, often showing comparable performance than BTE devices. It should be recognised that these variables may influence the comparisons.

#### Speech perception in quiet

Speech perception in quiet was tested by presenting the speech signal from the front (S0) in all studies. One study [15] reported no significant difference in performance between OTE (Kanso-1) and BTE (N5). Although there was no significant difference, a mean difference of 2.52% for OTE (Kanso-1) was reported in the Turkish CNC test compared with BTE (N6) [4]. Another study [18] reported no substantial difference in speech perception between the two processor designs (N6 and Kanso-1). Two studies [17, 21] indicated no significant differences in the FMT for OTE (Rondo-1) vs BTE (Opus-2) and in Australian CNC words for OTE (Kanso-1) vs BTE (N6), while comparable results in CUNY tests were found with BTE (Duet) vs OTE (Rondo-1) [20]. Two studies [19, 16] did not report performances in quiet conditions. Interestingly, one study [22] compared the first-generation OTE (Kanso-1) with OTE (Kanso-2) with ForwardFocus ON and found that speech understanding in quiet conditions was matched or improved with ForwardFocus ON in the Kanso-2 compared to the Kanso-1. This highlights the impact of processor generation on speech outcomes and provides caveats for comparisons. However, no comparisons were made between OTE and BTE in quiet conditions in this study. Across studies, findings suggest that OTE and BTE processors provide comparable performance for speech perception in quiet.

#### Speech perception in noise

Speech testing in noisy conditions were varied among the studies. The presentation conditions reported on were as follows; S0N0(speech and noise from front), S0NIL(speech from front and noise form ipsilateral side of CI), S0NCL(speech from front and noise from contralateral side of CI), S0N180 (speech from front and noise from back), S180N180 (speech and noise from back).Further, S0N3(speech from the front and 4-talker babble noise from 3 speakers equal to 12-talker babble), S0Nrearhemi (speech from front and 4-independent talkers presented from four locations roving across 7-speakers in the rear hemispheric field).

In S0N0 conditions, where noise and speech were at fixed levels, a significant difference was observed, with OTE (Kanso-1) outperforming BTE (N5) by a mean improvement of 8.1% in sentence scores [15]. Two studies [4, 20] reported no significant mean difference for OTE (Kanso-1) in the Turkish CNC test compared with BTE (N6) and in BKB-SIN tests with BTE (Duet) vs OTE (Rondo-1). In adaptive speech test conditions, where noise is fixed and the speech signal varies, significant improvements for OTE (Kanso-1) over BTE (N5) were reported [15]. However, three studies [4, 16, 19] reported no significant differences in speech scores between OTE and BTE. Notably, in one study [17], the exact placement of the loudspeakers was not clearly described. However, based on the reported results, it is assumed that both the speech and noise were likely coming from the same direction (S0N0), with comparable performance reported for OTE and BTE.

In conditions such as S0NIL, S0NCL, S0N180, and S180N180, mixed responses were reported. One study [4] found that in S0NCL conditions, performance scores were better for both OTE and BTE, with a significant impact on S0N180 and S180N180 conditions for both processors. Another study [18] reported better performance for OTE when switched to adaptive directionality in S0NIL, S0NCL, and S0N180 conditions but did not report on S180N180 conditions. Additionally, one study [19] reported a significant deterioration in performance with OTE (Rondo-1) in S0N180 conditions with no significant effect on S0NIL or S0NCL conditions.

S0N3 and S0Nrearhemi conditions were reported in two studies [21, 22]. Both studies showed an advantage for BTE over OTE during roving test conditions. Additionally, with ForwardFocus enabled for OTE (Kanso-2) and BTE (N7), a statistically significant benefit of 2.3 dB was observed for BTE over OTE [22]. When comparing processor types within Zoom, Beam, and SCAN settings, BTE (N6) performed better than OTE (Kanso-1), with a mean difference in SRT of 1.4 dB, 2.0 dB, and 1.4 dB, respectively.

Only three studies reported sound field aided threshold (SFAT) measurements. Two studies [16, 18] found no significant effect of OTE or BTE speech processors on the perception of the quietest sound in SFAT measurements. However, one study [4] reported that BTE (N6) performed better than OTE (Kanso-1) in SFAT measurements with a difference of 3.3 dB HL. It should be noted that, of the nine studies included in this review, only three studies reported SFAT results, which limits the generalizability of these findings and interpretations.

The findings from these studies suggest that OTE processors exhibit comparable or superior performance in both quiet and noisy conditions.

### Non-audiological outcomes

#### Medical complications

Limited data on medical complications associated with BTE and OTE was reported. One study [17] reported instances of redness, irritation, itching, and pain with OTE devices. No other significant medical complications were highlighted in the available data.

#### Device handling

Across multiple studies [16, 17, 20–22], recipients consistently favoured OTE processors in terms of device handling. Four studies [4, 15, 18, 19] did not comment on device handling in their outcome measures.

#### Retention

Only two studies documented the retention aspect of OTE processors through subjective ratings or user feedback questionnaires. One study [21] indicated that their cohort did not report any retention concerns. In contrast, another study [16] reported that 44% of their recipients experienced significant retention concerns at least once per day. However, none of the studies provided data logging reports regarding the number of unlock events or instances of the processor falling from the head.

#### Comfort

User comfort was a recurring finding favouring OTE over BTE across the five studies reported [16, 17, 20–22].

#### Self-assessment and overall satisfaction

Overall satisfaction, including a holistic assessment of device performance and user experience, leaned toward favouring OTE processors. Three studies [16, 20, 21] consistently reported recipients expressing higher satisfaction levels with OTE devices, reflecting a wider preference for this design across various measures. However, one study [17] reported that recipients were satisfied with OTE but did not dislike BTE processors. Another study [22] reported that some recipients felt negatively impacted by OTE and preferred to return to their BTE processors.

Studies reported self-assessment of perceived quality of life changes with several questionnaires, including HISQUI (hearing implant sound quality index), SSQ (speech, spatial, and qualities of hearing scale), and SHQ (spatial hearing questionnaire). Three studies reported these measures [16, 17, 20]. HISQUI [17] and SSQ [20] measured separately indicated similarity between OTE and BTE processors. However, one study [16] measured a collective assessment of HISQUI, SSQ, and SHQ and revealed superior outcomes with OTE over BTE recipients.

#### Quality of studies

Eight studies were of good quality with one being fair (high risk). The main concern was the acclimatization period used for the studies between the two processors. Most of the studies engaged the use of both processors, either in the same session or being relatively short period of usage. Three studies did not report on details, such as whether follow-up was long enough for outcomes to occur or how outcomes were assessed [15, 17, 19], while two studies [16, 20] lacked information on the selection of a non-exposed group. This again limits the effectiveness of follow up. Selection bias was apparent in two studies due to their limitation of sample and control groups. The quality assessment of all studies is summarized in Table 3.

**Table 3:** Quality and risk of bias assessment (Newcastle–Ottawa Scale) criteria.

Author	Selection				Comparability	Outcome				Total quality score	
	Representativeness of the exposed cohort	Selection of the non-exposed cohort	Ascertainment of exposure	Demonstration that outcome of interest was not present at start of study	Comparability of cohorts on the basis of the design or analysis	Assessment of outcome	Was follow-up long enough for outcomes to occur	Adequacy of follow up of cohorts			
**Jones et al. 2023 [22]**	*	*	*	*	*	*	**-**	*	**7**		Good
**Pinheiro et al .2021 [15]**	*	*	*	*	-	*	**-**	*	**6**		Fair
**Bayri et al. 2020 [4]**	*	*	*	*	*	*	**-**	*	**7**		Good
**Wesarg et al.2018 [18]**	*	*	*	*	*	*	**-**	*	**7**		Good
**Dazert et al. 2017 [17]**	*	*	*	*	-	-	-	*	**5**		Fair
**Mauger et al. 2017 [21]**	*	*	*	*	*	*	*	*	**8**		Good
**Wimmer et al .2015 [19]**	*	*	*	*	*	*	-	-	**6**		Fair
**Távora-Vieira et al. 2015 [20]**	**-**	**-**	**-**	*	*	*	*	*	**5**		Fair
**Mertens et al. 2014 [16]**	**-**	**-**	*	*	*	*	*	*	**6**		Fair

*Note*: NOS has a total maximum score of 9: Maximum scores 4 in Selection, 2 in Comparability, 3 in Outcome. Studies score from 7–9 have good quality (high quality), 4–6 have fair quality (high risk), and 0–3 have poor quality (very high risk). The symbol (*) means the point earned in each category and (-) no points

## Discussion

The review aimed to compare OTE and BTE sound processor devices for CI recipients. Key themes across studies were speech performance in quiet and noisy conditions, alongside non-audiological factors influencing performance and selection. These findings hold significance for guiding speech processor selection and enhancing communication outcomes for individuals with CIs.

### Audiological factors

#### Speech perception in quiet

Included studies indicate that performance in quiet conditions appears relatively comparable between OTE and BTE processors for CI recipients. However, understanding their efficacy in different listening environments is crucial. The majority of studies indicate no significant difference between OTE and BTE processors in quiet settings [4, 17, 18, 21]. Insights from additional studies consistently indicate comparable speech performance in quiet conditions, suggesting that changes in processor placement, from the pinna to the side of the skull, do not significantly impact speech perception in quiet conditions. The comparable performance also highlights improved microphone technology, enabling these devices to detect low-level input signals with fidelity. One study [22] further highlighted that with the latest OTE (Kanso-2) processors, even disabling certain user-controlled features, such as ForwardFocus options, does not degrade speech perception in quiet. It should be noted that the first generation OTE devices did show degraded performance. While performance in quiet conditions is comparable, these observations may not hold true in noisy or other challenging situations [21].

#### Speech perception in noise

It is well recognized that CI recipients find it hard to hear in noisy and challenging conditions. Background noise renders speech understanding even more difficult [22]. Regarding speech processor placement and performance in noisy situations, the included studies showed mixed results. One study [18] explored OTE processors with adaptive microphone directionality, providing important insights by indicating improved performance in noise with adaptive features turned on.

Similarly, one study [17] compared OTE (Rondo-1) and BTE (Opus-2) processors, challenging assumptions favouring BTE devices in noisy environments and demonstrating no loss in speech understanding with OTE processor placement. However, these findings should be interpreted cautiously considering the test environment setup and the specific research questions addressed. In contrast, one study [19] examined speech intelligibility in noise with OTE (Rondo-1) and reported that the processor positioned further behind the ear may affect speech perception in challenging acoustic conditions. Furthermore, another study [16] upgraded BTE to OTE for subjects with single-sided deafness and reported that the OTE processor position did not influence outcome measures. In fact, after a period of usage, the majority of the recipients accepted continued use of the OTE device.

The variability in performance observed across studies suggests that the effectiveness of OTE versus BTE processors can be influenced by several factors, including the directionality of noise and specific processor features. For instance, studies [4, 15, 16, 19] that evaluated fixed conditions with speech and noise from the front (S0N0) found mixed results. One study [15] reported a notable advantage for OTE (Kanso-1) over BTE (N5) in sentence scores. As mentioned before, caution must be applied in interpreting these effects based on the generation of processors being compared. For example, one study [15] compared two different generations of speech processor technologies: OTE (Kanso-1) vs BTE (N5). Kanso-1, with SCAN technology and automatic microphone directionality, offered better performance in various conditions compared to N5, which has two omnidirectional microphones.

In more dynamic and challenging noise conditions, such as those where noise is presented from different angles relative to speech (S0NIL, S0NCL, S0N180, and S180N180), the results are less consistent [4, 18]. This variability may reflect the differing abilities of OTE and BTE processors to handle complex noise environments. For instance, adaptive directionality features in OTE processors showed improved performance in certain conditions, suggesting that these features can enhance speech perception in noise for this type of processor.

However, other studies highlighted that BTE devices outperform OTE processors in specific noise configurations, such as S0N180 [19], as well as in roving conditions [21, 22]. The advantage of BTE devices in these conditions suggests they might offer superior performance in highly challenging listening scenarios.

#### Speech test set-up and performance outcomes

It must be noted that test set-up and processor technology can impact speech performance scores. As mentioned above, OTEs with dual microphones have shown improved performance scores compared to older generations of speech processing technology [4, 15, 22]. Furthermore, the dual microphone effect is thought to improve the SNR with OTE [4, 15]. Poor performance by OTE and to an extent with BTE in roving conditions still reinforces the need for continuous technological advances in segregating speech from competing environments.

Technologies such as ForwardFocus further improve speech intelligibility by emphasizing sounds coming from the front and reducing noise from other directions. This directional processing can be particularly beneficial in situations where the listener is facing the speaker, helping to enhance the clarity of the target speech [22].

Overall, these findings underscore the complexity of speech perception in noisy environments and suggest that while OTE devices offer competitive performance, BTE processors may have advantages in certain conditions, depending on the experimental set-up. Adaptive features in newer OTE processors (Kanso-2, Rondo-3) provide benefits but do not universally perform as well as BTE devices in all noisy scenarios. Continued research is essential to further understand these relationships and guide optimal CI device selection based on specific listening environments.

Despite this, improved processor technology in newer generations of OTE devices may offer comparable performance, which needs to be further investigated in future studies. For example, the current review did not include any studies comparing the latest BTE devices (Sonnet-2) with newer OTE processors (Rondo-3). It is possible that these newer OTE devices may perform comparably or even be superior to BTE devices, but this requires empirical validation. A comparison of BTE (N8) with OTE (Kanso-2) may already be outdated as technology marches forward.

#### Sound Field Aided Threshold (SFAT)

The limited number of studies reporting SFAT measurements indicates a need for further research. While some studies found no significant difference in SFAT performance between OTE and BTE, others reported better performance for BTE. This discrepancy highlights the necessity for more comprehensive research to understand the impact of processor type on perception not only in the speech testing condition but also for aided thresholds.

#### Effect of microphone placement

The positioning of microphones varies between the two sound processor designs, which is thought to impact speech understanding with OTEs. Findings from the included studies showed comparable speech understanding performance for both sound processor types across different positions in noise [4, 16, 18, 22]. Despite distinct microphone locations for BTE and OTE, the incorporation of dual-microphone features in both types of processors significantly reduced differences, yielding similar outcomes in various noisy conditions [18, 22]. Additionally, the uniformity in speech processing technology, microphone design, and background noise reduction algorithms across both sound processor types may contribute to the similar findings [21, 22].

Evidence from these studies indicate that speech perception in quiet and noise is highly influenced by factors including type of speech processor, microphone technology used, and other noise reduction features incorporated into the processors.

### Non-audiological factors

#### Device handling

Device handling is a critical non-audiological factor that influences the daily experience of CI recipients. Studies [16, 17, 20–22] comparing different speech processors provide insights into how variations in device design and functionality impact user interactions. Particularly in ease of use for telephone calls and wearing glasses, OTE was preferred over BTE [17], but in cases of SSD, subjects preferred to use the contralateral normal hearing ear for telephone usage [16]. Other studies did not report recipient feedback on telephone usage with OTE versus BTE. It must be noted that OTEs reported in this review did not offer the Bluetooth streaming option for telephones, although newer OTE models are now available with Bluetooth streaming capabilities. Understanding the ergonomics and user-friendliness of CI processors, as revealed by these studies, is essential for optimizing the handling experience and ensuring ease of use for individuals with CI.

#### Retention

Retention of CI processors is crucial for user confidence and uninterrupted auditory experiences, especially with the OTE lacking surrounding structures for easy anchoring [23]. One study [16] reported that OTE processors unexpectedly fall off in daily use. Contrary to this, another study [21] found no significant difference in retention between OTE and BTE. Interestingly, some recipients’ initial reliance on retention accessories for OTE decreased over time as users gained confidence. Some recipients felt superior retention with OTE processors in specific situations, such as lying down or bending over, which may provide potential advantages in certain real-world scenarios [4, 21, 22]. These insights into device retention are valuable, guiding considerations for recipients and clinicians concerning device stability and security during daily activities.

#### Comfort

Comfort plays a significant role in the long-term acceptance and usability of CI devices. Comfort and retention of OTE processors depend on selected magnet strength, which is influenced by factors such as skin flap thickness and specific retention requirements [16, 21]. To improve comfort, manufacturers provide soft pads to attach beneath the processor, distributing pressure across the processor area and increasing coil-implant separation. Clinical outcomes with OTE processors combined with user comfort help explain how device design and form factor impact the overall wearing experience [21]. Wearing comfort for OTE was further enhanced when used with spectacles compared to BTEs, as well as offering improved comfort over tight-fitting ear moulds with BTEs [16, 17, 20]. These findings reinforce the principle that processor comfort preferences are vital for long-term satisfaction.

#### Medical complications

Although not an explicitly stated outcome in the primary studies, only one study reported complications related to OTE devices, such as infections, redness, irritation, itching, and pain over the implant site [17]. Often, these issues could be related to the increased magnet strength chosen for recipients, which clinicians could mitigate. As previously mentioned, skin flap thickness can impact the risk of skin-related irritations with OTE. These issues are also observed in BTE processors, although to a lesser extent.

#### Overall satisfaction

Overall satisfaction with OTE compared to BTE encompasses varied aspects of the CI experience. One study [17] reported that more than 70% of participants would highly recommend OTE over BTE after six months of using the processors. Another study [16] reported that 80% of participants favoured the OTE over their previous BTE device after 28 days of usage. Therefore, overall satisfaction depends on a holistic approach to CI assessments, considering not only functional outcomes but also user preferences and satisfaction [20–22].

#### Effect of device configurations

The head-shadow and binaural squelch effects are particularly relevant in noisy environments, where spatial separation between target speech and competing sounds significantly enhances speech understanding. Evidence from the included studies indicates that bilateral CIs are superior, especially in noisy and context-dependent situations, due to the substantial binaural benefits they offer [15]. Bilateral cues enhance speech intelligibility and daily life performance more effectively than unilateral use, which often shows poorer results due to the lack of stimulus redundancy. There were no or limited comparisons reported between BTE and OTE under bimodal configurations, limiting the inferences that could be drawn from the available evidence. Only one study [20] reported on the Electroacoustic System (EAS), comparing BTE and OTE with ITE, and found a preference among subjects for OTE over BTE.

Though there is no evidence-based guidance on the use of OTE or BTE from this review, it is essential to tailor recommendations based on the individual needs and circumstances of each recipient when considering different device configurations, such as OTE or BTE in bilateral, unilateral, and bimodal setups. These configurations can significantly impact auditory outcomes and user experiences.

#### Sound processor technology

The sound processors included in the review were both BTE and OTE processors from MED-EL® and Cochlear® Ltd, including models like BTE-N7, N6, N5, Opus2, Duet, and OTE-Kanso-1, Kanso-2, Rondo-1. These processors lack some technological advances available to the CI recipients at the time of writing this review, such as hands-free options, enhanced usage in challenging environments, and improved telephone usability, although we have discussed ForwardFocus options for the Kanso-2 OTE.

The authors note that no studies have compared the latest processor technology, such as N8 (BTE) with Kanso-2 (OTE) as well as Sonnet-2 (BTE) with that of Rondo-3, and Rondo-2 (OTE). These newer OTE and BTE models offer significant technological advances, including improved speech processing algorithms and non-audiological factors like lighter weight processors, better retention and Bluetooth streaming options. Empirical evidence in these areas would enhance clinical knowledge and improve recipient satisfaction.

### Clinical implications

Findings from the reviewed studies indicate comparable speech performance between OTE and BTE speech processors in various environments. With the implementation of modern dual microphone technologies into OTE devices, spatial separation from noise and other standard directionality can be achieved. Despite prior BTE experience, most of the participants in the review preferred OTE over BTE with significantly higher comfort, aesthetics, ease of use, and overall hearing performance. These findings suggest that individuals needing discretion and simplicity, OTE processor may be an alternative to traditional BTE processors. It must be noted that OTE is not the ideal solution for every potential CI user and some recipients may still prefer BTE. It is important in clinical settings that this option should be discussed during CI candidacy assessment as well as during planned CI processor upgrades.

### Limitations

A key limitation of the review is that most of the studies reported in the review used the first generation of OTE processors, except one study reporting on the Kanso-2 OTE [22]. This potentially underestimates the effects of OTE technology on outcomes as improved OTE processors are now available for CI recipients. BTE devices have also undergone improvements in sound processing technologies since the earlier generation devices. The modest methodological quality and heterogeneity among the reviewed studies precluded meta-analysis. The varied acclimatization period, especially with processor change in same session, may subjectively incline the recipients for OTE over BTE.

### Future scope of research

Future research may compare different speech processing strategies with OTE and BTE and further focus on the durability and ease of use and retention with more recently available, lighter processors, and with new magnet technologies, have on CI recipients. More controlled studies on the microphone placement (i.e. how far back the OTE devices are placed) and how it interacts with real world performance may be future work that can be undertaken by manufacturers or clinicians. Also, it is important to examine the advantages of OTE in relation to recipient’s overall lifestyle, daily activities, use of wireless connectivity and accessories may enhance the evidence base for counselling.

## Conclusion

The findings of this review indicate that OTE processors exhibit comparable or superior performance in both audiological and non-audiological aspects of device usage. This systematic comparison between BTE and OTE processors helps clinicians to make informed recommendations, tailoring the choice of design to individual patient requirements. Furthermore, manufacturers can utilise these findings to enhance and advance future CI processors. The implications of this review underscore the significance of considering OTE processors as a viable and potentially advantageous option for CI recipients. Some caution should be exercised as OTE processors may exhibit retention concerns and not all CI recipients are ideal candidates for OTE processors; some may still prefer to use BTE.

## Supporting information

S1 File Preferred Reporting Items for Systematic reviews and Meta-Analyses (PRISMA-S) checklist. (DOCX)

S2 File Search strategy. (DOCX)

S3 File Description of the records screened. (XLSX)

## Abbreviations

A-CNC: Australian consonant-nucleus-consonant
AuSTIN: Australian Speech Test In Noise
BTE: behind the ear
BKB-SIN: Bamford-Kowal-Bench- Speech in noise
OST: Oldenburg Sentence Test
OTE: off the ear
CI: cochlear implant
CUNY: City University of New York
FMT: Freiburg Monosyllables test
HINT: Hearing in Noise Test
HISQUI: Hearing Implant Sound Quality Index
LIST: Leuven Intelligibility Sentences Test
NOS: Newcastle-Ottawa Scale
SHQ: Spatial Hearing Questionnaire
SPEQ: Sound Processor Experience Questionnaire
SSQ: Speech, Spatial, and Qualities
T-CNC: Turkish consonant-nucleus-consonant
TMT: Turkish Matrix test
