# Supplementary material for: Comparison of behind-the-ear vs. off-the-ear speech processors in cochlear implants: A systematic review and narrative synthesis
Source: PLoS One. 2025 Jan 27;20(1):e0318218. doi: 10.1371/journal.pone.0318218 (PMC11771919; doi:10.1371/journal.pone.0318218)
Supplement: S2 File — (DOCX) [file pone.0318218.s002.docx]

**Supplementary material 1**

Data bases searches

**Cochrane Library**

((cochlea* or auditory) Near/5 (implant* or prosthe*)):ti,ab,kw OR MeSH descriptor: [Cochlear Implants] this term only or MeSH descriptor: [Cochlear Implantation] this term only

AND

("speech process*" or "sound process*"):ti,ab,kw. or MeSH descriptor: [signal processing, computer assisted] explode all trees

AND

("off the ear*" or OTE or ("behind the ear*" or BTE) or (microphone location or (entrance near/4 canal) or (top near/4 pinna))):ti,ab,kw.

**Scopus**

Title-Abs-Key ((cochlea* or auditory) W/5 (implant* or prosthe*))

AND

Title-Abs-Key ("speech process*" or "sound process*")

AND

Title-Abs-Key ("off the ear*" or OTE or ("behind the ear*" or BTE) or (microphone location or (entrance w/4 canal) or (top w/4 pinna)))

**ProQuest Dissertations & Theses A&I**

abstract((cochlea* or auditory) near/5 (implant* or prosthe*)) or title ((cochlea* or auditory) near/5 (implant* or prosthe*)) or Exact("cochlear implants")

AND

Abstract("speech process*" or "sound process*") or title("speech process*" or "sound process*")

AND

Abstract("off the ear*" or OTE or ("behind the ear*" or BTE) or (microphone location or (entrance near/4 canal) or (top near/4 pinna))) or title("off the ear*" or OTE or ("behind the ear*" or BTE) or (microphone location or (entrance near/4 canal) or (top near/4 pinna)))
